# Differential sensitivity of cinnamaldehyde-evoked calcium fluxes to ruthenium red in guinea pig and mouse trigeminal sensory neurons

**DOI:** 10.1186/s13104-021-05539-2

**Published:** 2021-04-07

**Authors:** Parmvir K. Bahia, Thomas E. Taylor-Clark

**Affiliations:** Molecular Pharmacology & Physiology, Morsani College of Medicine, University of South Florida, 12901 Bruce B Downs Blvd, Tampa, FL 33612 USA

**Keywords:** TRPA1, Trigeminal, Ruthenium red, Guinea pig, Mouse

## Abstract

**Objective:**

Transient receptor potential ankyrin 1 (TRPA1) is an excitatory ion channel expressed on a subset of sensory neurons. TRPA1 is activated by a host of noxious stimuli including pollutants, irritants, oxidative stress and inflammation, and is thought to play an important role in nociception and pain perception. TRPA1 is therefore a therapeutic target for diseases with nociceptive sensory signaling components. TRPA1 orthologs have been shown to have differential sensitivity to certain ligands. Cinnamaldehyde has previously been shown to activate sensory neurons via the selective gating of TRPA1. Here, we tested the sensitivity of cinnamaldehyde-evoked responses in mouse and guinea pig sensory neurons to the pore blocker ruthenium red (RuR).

**Results:**

Cinnamaldehyde, the canonical TRPA1-selective agonist, caused robust calcium fluxes in trigeminal neurons dissociated from both mice and guinea pigs. RuR effectively inhibited cinnamaldehyde-evoked responses in mouse neurons at 30 nM, with complete block seen with 3 μM. In contrast, responses in guinea pig neurons were only partially inhibited by 3 μM RuR. We conclude that RuR has a decreased affinity for guinea pig TRPA1 compared to mouse TRPA1. This study provides further evidence of differences in ligand affinity for TRPA1 in animal models relevant for drug development.

## Introduction

Transient Receptor Potential Ankyrin 1 (TRPA1) is a homo-tetrameric, non-selective cation channel belonging to the superfamily of TRP channels. Mammalian TRPA1 is expressed primarily in a subset (~ 20–50%) of nociceptive sensory nerves, with cell bodies in dorsal root ganglia (DRG), trigeminal ganglia, and vagal ganglia [1, 2]. TRPA1 is activated by a range of electrophilic chemical irritants and products of oxidative stress, and plays an important role in initiating or sensitizing noxious sensations and nocifensive reflexes in the skin and visceral organs such as the airways [3–7]. As such, TRPA1 has been proposed as a target for therapeutic intervention in the treatment of pain and respiratory disorders.

TRPA1 have been studied using recombinant channels and various animal models. With respect to respiratory physiology, activation of TRPA1 by irritants, oxidative stress and pollutants causes cough, apnea, reflex bronchospasm and reflex modulation of heart rate [6, 8–14]. Furthermore, knockout of TRPA1 has been shown to be protective in allergen-associated airway hyperreactivity [15]. The guinea pig (*Cavia porcellus*) is of particular interest for developing therapeutic targets for respiratory diseases as airway smooth muscle physiology, acute responses to allergen exposure, and ability to cough in response to inhaled irritant aerosols are more representative of human airways than those of rats or mice [16, 17]. However, despite the many studies examining TRPA1 function in guinea pig tissues, the characterization of the channel itself remains incomplete. This is an important deficit to address given the marked differences in amino acid sequence and functional disparities between rodent and human TRPA1 [18, 19].

One of the most commonly used pharmacological tools in the study of cation channels is the non-specific blocker Ruthenium Red (RuR). This water soluble, inorganic cationic dye was shown by Moore in 1971 [20] to inhibit transmembrane calcium fluxes, and has since been demonstrated as an effective blocker of calcium channels and, in particular, TRP channels [1, 21–23]. RuR has been shown to block the outer pore of different TRP channels and is thought to bind to specific aspartic acid residues [24, 25]. In the case of heterologously-expressed TRPA1, RuR has been shown to block both mammalian and non-mammalian orthologs of the channel [3–5, 26–28] and, in accordance with other TRP channels, this block is greatly diminished in human TRPA1 if the D915 residue is mutated [29]. There has been a report that heterologously-expressed guinea pig TRPA1 is less sensitive to RuR block compared to heterologously-expressed mouse TRPA1 [30]. Here we have investigated the effect of RuR on the activation of trigeminal neurons from mice and guinea pigs by the electrophilic TRPA1 agonist cinnamaldehyde. Previous studies have shown that cinnamaldehyde-evoked sensory nerve responses in rodents are largely eliminated by TRPA1 knockout or selective TRPA1 inhibitors [9, 31–34]. We found marked differences in RuR’s inhibition of cinnamaldehyde-evoked responses, with effective block of mouse responses at 30 nM but only partial block of guinea pig responses at 3 μM. We conclude that guinea pig TRPA1 is less sensitive to RuR block compared to mouse TRPA1.

## Main text

### Methods and results

To study the differences between the sensitivity of guinea pig TRPA1 and mouse TRPA1 to RuR we compared its effect on the Ca^2+^ responses of sensory neurons dissociated from mouse and guinea pig trigeminal ganglia to the TRPA1 selective agonist cinnamaldehyde [3, 34]. We have previously shown that TRPA1 agonists increase [Ca^2+^]_i_ by inducing Ca^2+^ influx [35, 36]. Male C57BL/6 mice (6 weeks old, 6 animals, purchased from Envigo) and male Hartley guinea pigs (6 weeks old, 3 animals, purchased from Charles River) were killed by CO_2_ asphyxiation followed by exsanguination. Trigeminal ganglia were immediately isolated and enzymatically dissociated using previously described methods [5]. Isolated neurons were plated onto poly-D-lysine and laminin-coated coverslips, incubated at 37 °C in L-15 (supplemented with 10% fetal bovine serum) and used within 24 h. Neurons were studied for changes in [Ca^2+^]_i_ with Fura-2AM, as before [36]. Neuron-covered coverslips were incubated (37 °C) with Fura-2AM (4 μM, for 30 min) in L-15 media containing 10% fetal bovine serum. For imaging, the coverslip was placed in a custom-built heated chamber and superfused HEPES-buffered bath solution (composition (mM)): 154 NaCl, 4.7 KCl, 1.2 MgCl_2_, 2.5 CaCl_2_, 10 HEPES, 5.6 dextrose adjusted to pH 7.4 with NaOH) for 10 min before and throughout each experiment. Changes in [Ca^2+^]_i_ were monitored by sequential dual excitation, 340 and 380 nm (emission 510 nm)(CoolSnap HQ2; Photometrics, Surrey, BC, Canada) and analyzed by Nikon Elements (Nikon, Melville, NY, USA). Neurons were exposed to cinnamaldehyde (50 μM) at t = 4 to 6 min, then, following a washout of 7 min, to a second cinnamaldehyde treatment (100 μM) at t = 13–15 min. Neurons were treated with various concentrations of ruthenium red (0, 30, 300, 3000 nM) preceding, during and after the first cinnamaldehyde treatment at t = 1–8 min. At the end of the studies, all neurons were exposed to KCl (75 mM, 60 s) to confirm voltage sensitivity and ionomycin (5 μM, 60 s) to obtain a maximal response. All agents were purchased from Sigma-Aldrich.

As before [37], we determined [Ca^2+^]_i_ by measuring the 340/380 ratio and relating all measurements to the peak positive response in each cell. Thus we have chosen to normalize ratiometric responses at each timepoint in each cell to its maximum [Ca^2+^]_i_ (evoked by the Ca^2+^ ionophore ionomycin) – data was presented as the percentage change in 340/380 ratio (R): response at time (x) = 100 X (R_x_-R_bl_)/(R_max_-R_bl_), where R_x_ was the 340/380 ratio of the cell at a given time point, R_bl_ was the cell’s mean baseline 340/380 ratio measured over 60 s, and R_max_ was the cell’s peak 340/380 ratio. Only cells that had low [Ca^2+^]_i_ at baseline (R < 1.0) and yielded a robust response to the positive control were included in analyses. Neurons were defined as TRPA1-expressing if a positive response was noted for either cinnamaldehyde treatments. Only cinnamaldehyde-sensitive neurons were included in the analyses. Ratiometric responses were found to pass the D’Agostino & Pearson test for normality (p > 0.05). The concentration response graph represents unpaired observations, which were compared using a 2-way ANOVA with Dunnett’s multiple comparisons using GraphPad Prism version 8. A p value less than 0.05 was taken as statistically significant.

**Fig. 1:**
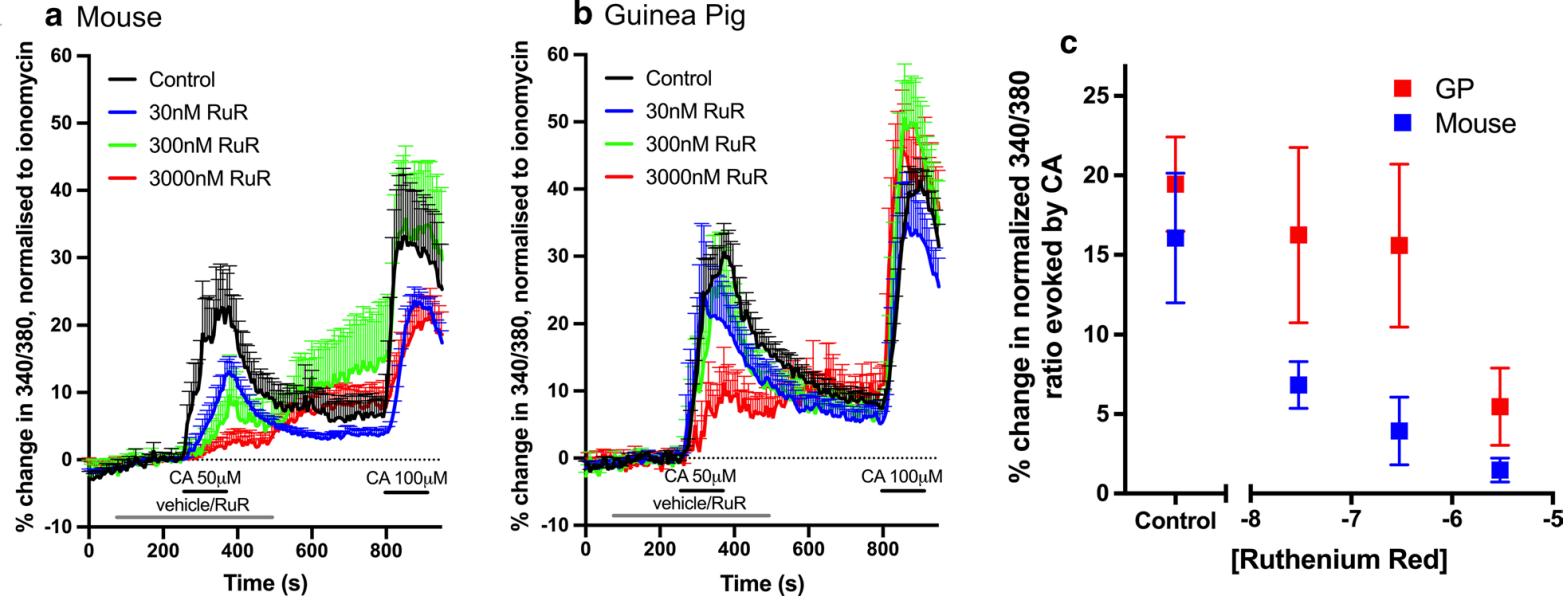
Inhibition of cinnamaldehyde-evoked responses by ruthenium red (RuR) in mouse and guinea pig trigeminal neurons. *A and B,* mean (± 95% confidence intervals) [Ca^2+^]_i_ responses of mouse (**a**) and guinea pig (**b**) trigeminal neurons to cinnamaldehyde (CA, 50 μM cinnamaldehyde) in the presence of vehicle, or RuR (30 nM to 3 µM). After RuR washout, 100 µM cinnamaldehyde was applied as a positive control for TRPA1 expression. *C,* concentration response relationship for RuR in inhibiting the mean (± 95% confidence intervals) [Ca^2+^]_i_ responses evoked by 50 μM cinnamaldehyde (CA) in mouse (blue squares) and guinea pig (GP, red squares) trigeminal neurons. N = 27, 57, 21 and 47 mouse neurons for vehicle, 30 nM, 300 nM, or 3000 nM, respectively. RuR. N = 134, 34, 45 and 53 guinea pig neurons for vehicle, 30 nM, 300 nM, or 3000 nM RuR, respectively

Cinnamaldehyde (50 μM and 100 μM) caused an increase in [Ca^2+^]_i_ in a subset of trigeminal neurons from both mouse (152 out of 560 neurons, 27%) and guinea pig (266 out of 571 neurons, 47%) (Fig. 1a, b). RuR inhibited the calcium responses evoked by 50 μM cinnamaldehyde in mouse trigeminal neurons at 30 nM, with complete block seen with 3 μM (Fig. 1a). In contrast, 50 μM cinnamaldehyde evoked robust calcium responses in guinea pig trigeminal neurons even in the presence of 300 nM RuR, and responses were only partially inhibited by 3 μM RuR (Fig. 1b). Inhibition of cinnamaldehyde-evoked responses by RuR was reversible in both mouse and guinea pig neurons (Fig. 1a, b). Consistent with previous studies [24, 38] RuR’s block washed out rapidly, in some neurons exposing latent cinnamaldehyde-evoked responses. Plotting the concentration–response relationship of RuR’s inhibition of cinnamaldehyde-evoked calcium responses showed that the TRPA1 blocking ability of RuR was greater in mouse neurons compared to guinea pig neurons (2-way ANOVA, p = 0.0002 for species comparison (F statistic (1, 410) = 14.3), p < 0.0001 for the effect of RuR concentration (F statistic (3, 410) = 22.14)), with RuR’s concentration–response relationship right shifted in guinea pig neurons (Fig. 1c). These data clearly demonstrate an inter-species difference in the sensitivity of cinnamaldehyde-evoked responses to this extensively used blocker.

### Discussion

Previous studies have shown that guinea pig and mouse nociceptive sensory nerves and nocifensive reflexes are activated by known TRPA1 agonists such as cinnamaldehyde and other unsaturated aldehydes and allyl isothiocyanate [3, 4, 8, 10, 11, 13, 39–41]. As expected, cinnamaldehyde here activated a subset of sensory neurons derived from both guinea pig and mouse trigeminal ganglia. However, the sensitivity of these cinnamaldehyde-evoked responses to RuR was substantially different between the two species: mouse cinnamaldehyde-evoked responses were robustly inhibited by 30 nM RuR, whereas guinea pig cinnamaldehyde-evoked responses were only partially blocked at 3 μM. Previous research has shown that cinnamaldehyde-evoked sensory nerve responses in rodents are largely eliminated by TRPA1 knockout or selective TRPA1 inhibitors [9, 31–34], thus it is likely that the cinnamaldehyde-evoked responses shown here are also mediated by TRPA1. Previous studies have shown that micromolar concentrations of RuR are capable of some degree of inhibition of TRPA1-mediated responses in neurons and tissues from guinea pigs [10, 34, 42] and mice [3, 5, 38]. Interestingly, there has been a report that heterologously-expressed guinea pig TRPA1 is less sensitive to RuR block compared to heterologously-expressed mouse TRPA1 [30]. But comparison concentration–response relationships of RuR’s inhibition of TRPA1-mediated responses in native guinea pig and mouse neurons have not previously been published. RuR blocks human TRPA1-mediated currents with an IC50 of 45 nM [29], which resembles the mouse data presented here. RuR is a positively charged pore blocker whose actions on human TRPA1 are dependent in part on D915 [29]. This residue is also present in both mouse and guinea pig TRPA1, thus it is likely that other pore residues are important for the differential sensitivity of guinea pig TRPA1 to RuR inhibition [30].

Guinea pigs are important animal models for respiratory diseases [16, 17]. Recent studies have highlighted the critical role that sensory nerves play in respiratory and cardiovascular diseases [43–45]. This study shows that TRPA1, which contributes to nociceptive signaling by pollutants, irritants, oxidative stress and inflammation [15, 46], has notable differences in its modulation by pharmacological agents in animal models commonly used for drug development [18, 19]. As such, caution is required in extrapolating ligand potentiates between species.

## Limitations

This study used cinnamaldehyde as a model selective agonist for TRPA1. Nevertheless, we acknowledge that, despite previous data demonstrating the TRPA1-selective responses of cinnamaldehyde [9, 31–34], we have not definitively demonstrated the specific involvement of TRPA1. This study compared cinnamaldehyde-evoked changes in [Ca^2+^]_I_, which is an indirect measure of TRPA1 channel activation. This technique was chosen because it is high throughput. More definitive data may be derived from direct assessment of TRPA1-mediated currents in low throughput whole cell patch clamp studies. In addition, these studies were performed on native neurons whose TRPA1 channel activities may be modulated by the expression of other proteins and pathways. Studies of heterologously-expressed cloned TRPA1 channels may reduce the likelihood of these potential confounding effects. Furthermore, mutations of cloned channels would add a mechanistic biophysical component to these studies. Nevertheless, the data presented here provides strong evidence that ruthenium red has a lower affinity for guinea pig TRPA1 compared to mouse TRPA1.

## Data Availability

The datasets used and analyzed during the current study are available from the corresponding author on reasonable request.

## Abbreviations

CA: Cinnamaldehyde
GP: Guinea pig
RuR: Ruthenium red
TRPA1: Transient receptor potential ankyrin 1
